# No *Wolbachia* infection was detected in *Drosophila elegans* collected from the wild in the Ryukyu Islands, Japan

**DOI:** 10.17912/micropub.biology.000644

**Published:** 2022-09-28

**Authors:** Masatoshi Tomaru, Toshiyuki Takano-Shimizu-Kouno, Haruka Wakada

**Affiliations:** 1 KYOTO Drosophila Stock Center and Faculty of Applied Biology, Kyoto Institute of Technology, Kyoto 616-8354, Japan; 2 Undergraduate Program of Integrated Science and Technology, School of Science and Technology, Kyoto Institute of Technology, Kyoto 606-8585, Japan

## Abstract

Flower breeding, tropical and subtropical *Drosophila elegans* is distributed in the Ryukyu Islands and Taiwan (black morph) and in southern China, Philippines, Indonesia, and New Guinea (brown morph). Although reproductive and behavioral manipulations by *Wolbachia* are reported in many insect taxa, *Wolbachia* infection in *D. elegans* is unclear. There is only a report of no *Wolbachia* detected in a laboratory strain of brown morph. This PCR diagnosis study revealed no *Wolbachia* infection in *D. elegans* males collected from the wild in the Ryukyu Islands. We concluded that *D. elegans* black morph in the Ryukyu Islands is not infected with *Wolbachia* .

**Figure 1. PCR analysis for mitochondrial 12S rRNA as a control (A, 379 bp fragments) and Wolbachia wsp gene (B, 601 bp fragments).**
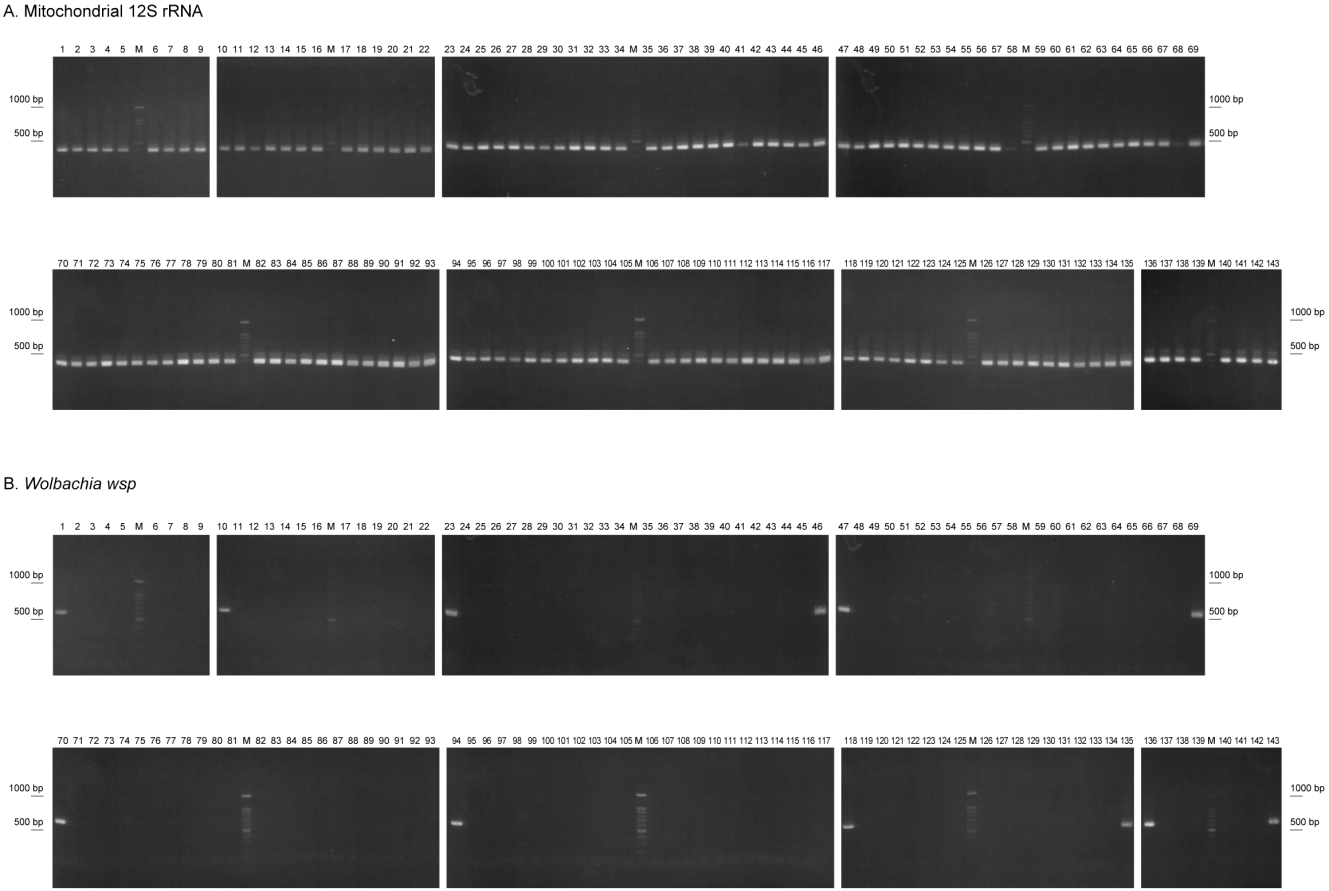
No fragment of *Wolbachia* *wsp* gene was amplified in *D. elegans* . See lane numbers in the Reagents section.

## Description

*Drosophila elegans* is a tropical and subtropical species distributed in southeast Asia and breeds on flowers of morning glory ( *Ipomoea* spp.), trumpet ( *Brugmansia candida* ) and turmeric ( *Cucurma domestica* ) (Okada and Carson 1982; Sultana et al. 1999; Yoshida et al. 2000). Natural host plants of *D. elegans* are *Ipomoea* in the Ryukyu Islands, Japan. A male of *D. elegans* stays singly on a flower and shows territorial behavior; when another male visits, he chases and kicks the competitor, and pushes him out of the flower. In contrast, when a female visits the flower to lay eggs, he courts her. Two morphs are found in this species: black morphs from the Ryukyu Islands and Taiwan and brown morphs from southern China, Philippines, Indonesia, and New Guinea (Bock and Wheeler 1972; Okada and Carson 1982; Lemeunier et al. 1986; Hirai and Kimura 1997). Reproductive and behavioral manipulation by *Wolbachia* is reported in many insect taxa, including *D. melanogaster* (Kose and Karr 1995; Riegler et al. 2005; De Crespigny et al. 2006). However, it is less clear whether *Wolbachia* infection affects reproduction and other biological processes in *D. elegans* . Although Bourtzis et al. (1996) reported no *Wolbachia* detection in a laboratory strain of brown morph of *D. elegans* that was collected from Baguio City, Luzon, Philippines, there is no attempt to detect *Wolbachia* in freshly collected *D. elegans* flies, in our literature search. The present study attempted to detect *Wolbachia* in *D. elegans* (black morph) freshly collected from the wild on Okinawajima Island, Iriomote Island and Ishigaki Island, the three largest islands of the Ryukyu Islands.

DNAs were extracted from single male flies and then performed on PCR diagnosis of *Wolbachia* detection. No PCR-amplified fragment of *Wolbachia wsp* gene was detected in 114 *D. elegans* males freshly collected (63 on Okinawajima Island, 33 on Iriomote Island and 18 on Ishigaki Island) from flowers of morning glory (Figure 1). We have established two independent lines from several collected males and females (“mass lines”). PCR diagnosis also showed no *Wolbachia* in males from these mass lines, approximately the 7th generation from the collected flies. In addition, no *wsp* fragment was amplified from two *D. elegans* stock-center strains (black morph) collected on Okinawajima Island and Iriomote Island in 2016 and 1993, respectively, and a brown morph strain collected in Hong Kong in 1993. In contrast, samples from *Wolbachia* -infected *D. mauritiana* , collected in 1979, showed amplified fragments, suggesting that *Wolbachia wsp* PCR primers worked to detect *Wolbachia* infection. PCR-amplified fragments of mitochondrial 12S rRNA were detected from all samples, indicating that 0.5 μl of DNA extracts from single males sufficiently contained genomic DNAs to be amplified by PCR. We repeated PCR amplification on a different day and again resulted in no detection of *Wolbachia* in *D. elegans* .

From these results, we concluded that there is no *Wolbachia* infection in *D. elegans* collected from the wild in this study. The collection sites for this study were the northeastern (Okinawajima Island) and southwestern islands (Iriomote Island and Ishigaki Island) of the Ryukyu Islands; the maximum distance between collection sites is approximately 540 km and we did not collect flies from other islands between these three islands. In *D. simulans* , *P* elements invaded more than 1,000 km along the Japan Archipelago within three years (Yoshitake et al. 2018). Although it is a different species from *D. elegans* , it is very likely that there are gene flows in *D. elegans* along the Ryukyu Islands. Therefore, it is likely that *D. elegans* in the Ryukyu Islands is not infected with *Wolbachia* .

## Methods

DNA extraction from a single male was as follows: a fly was homogenized in 50 μl of DNA extraction solution (10 mM Tris-HCL, pH 8.0/1 mM EDTA, pH 8.0/25 mM NaCl/20 μg/ml proteinase K/0.5% Tween-20), and incubated for 15 min at 45℃, followed by 10 min at 95℃. A 0.5 μl of DNA extract was used for PCR amplification with 1 μl of PCR primers for each (insect mitochondrial 12S rRNA primers for the controls to check for the quality of each DNA extraction (O’Neill et al. 1992), 379 bp of an expected fragment length or *Wolbachia wsp* gene primers (Braig et al. 1998, Jeyaprakash and Hoy 2000), 601 bp of an expected fragment length), 10 μl of PrimeSTAR MAX DNA Polymerase (Takara, Japan) and 7.5 μl of sterilized water. PCR amplification was made under the following conditions: 98℃ for 10 s, 30 cycles of 98℃ for 10 s, 55℃ for 5 s, and 72℃ for 3 s.

## Reagents

*Flies*

Using a mouth aspirator, we collected *D. elegans* male flies on morning glory ( *I. indica* ) flowers by the roadside, on the northern part of Okinawajima Island, the largest island in the Ryukyu Islands, the southwestern part of Japan, Iriomote Island, the second largest island and Ishigaki Island, the third largest island. The collection location, the collection date, and the ID numbers of the flies (Fly#s) which are the same as the lane numbers of the agarose gels in Figure 1 are as follows:

Flies collected on Okinawajima Island: [Ogimi Village, Kunigami County, Okinawa Prefecture, Japan] Fly# 11 and 12 (26°42'1"N 128°7'24"E, 22 March 2021), Fly# 31-39 (26°40'38"N 128°8'53"E, 26 April 2021), Fly# 40-45 and 48 (26°40'59"N 128°8'55"E, 26 April 2021), Fly# 49-52 (26°41'20"N 128°9'1"E, 26 April 2021). [Kunigami Village, Kunigami County, Okinawa Prefecture, Japan] Fly# 13 (26°46'6"N 128°12'17"E, 22 March 2021), Fly# 14-16 (26°52'2"N 128°15'10"E, 22 March 2021), Fly# 17-21 (26°51'57"N 128°15'21"E, 22 March 2021), Fly# 22 (26°51'57"N 128°15'21"E, 22 March 2021), Fly# 53-68 (26°52'2"N 128°15'10"E, 27 April 2021), Fly# 131 and 132 (26°52'2"N 128°15'10"E, 27 March 2022), and Fly# 133 (26°51'58"N 128°15'22"E, 27 March 2022). [Nago City, Okinawa Prefecture, Japan] Fly# 24-27 (26°37'36"N 128°3'26"E, 26 April 2021), Fly# 28-30 (26°37'40"N 128°3'29"E, 26 April 2021). [Motobu Town, Kunigami County, Okinawa Prefecture, Japan] Fly# 126-130 (26°40'54"N 127°53'0"E, 27 March 2022).

Flies collected on Iriomote Island (Taketomi Town, Yaeyama County, Okinawa Prefecture, Japan): Fly# 71 (24°25'37"N 123°46'44"E, 23 March 2022), Fly# 72-74 (24°15'40"N 123°51'36"E, 24 March 2022), Fly# 75-81 (24°15'39"N 123°50'6"E, 24 March 2022), Fly# 82-86 (24°18'4"N 123°52'42"E, 24 March 2022), Fly# 87 (24°25'38"N 123°46'44"E, 24 March 2022), Fly# 88-90 (24°22'50"N 123°54'38"E, 24 March 2022), Fly# 91, 92, 95-98 (24°22'42"N 123°54'53"E, 24 March 2022), Fly# 99-101 (24°22'33"N 123°55'1"E, 24 March 2022) and Fly# 102-105 (24°22'26"N 123°55'2"E, 24 March 2022).

Flies collected on Ishigaki Island (Ishigaki City, Okinawa Prefecture, Japan): Fly# 106-112 (24°21'21"N 124°8'2"E, 26 March 2022), Fly# 113 and 114 (24°21'48"N 124°7'15"E, 26 March 2022), Fly# 115, 116,119 and 120 (24°22'37"N 124°7'30"E, 26 March 2022), Fly# 121-124 (24°22'41"N 124°7'45"E, 26 March 2022) and Fly# 125 (24°22'55"N 124°8'3"E, 26 March 2022).

Mass lines originally collected on Okinawajima Island (Ogimi Village, Kunigami County, Okinawa Prefecture, Japan): Fly# 137-139 (26°40'38"N 128°8'53"E, 27 April 2021) and Fly# 140-142 (26°42'1"N 128°7'48"E, 27 April 2021).

Stock-center strains: *D. mauritiana* g-74 collected in Mauritius in 1979, a strain infected with *Wolbachia* (Ehime-Fly stock# E-18905) (Fly# 1, 10, 23, 46, 47, 69, 70, 94, 118, 135, 136 and 143). *D. melanogaster* Oregon-R, a strain not infected with *Wolbachia* : (Fly# 8, 9, 117, 134 and 143). *D. elegans* OKN16-1 collected in Okinawa, Japan in 2016 (Kyorin-Fly stock# E-13207) (Fly# 2 and 3), *D. elegans* IR-MTK collected on Iriomote Island, Okinawa, Japan in 1993 (Kyorin-Fly stock# E-13201) (Fly# 4 and 5), and *D. elegans* HKG-MTK collected in Hong Kong in 1993 (Kyorin-Fly stock# E-13202) (Fly# 6 and 7).

*PCR primers*

Insect mitochondrial 12S rRNA primers (O’Neill et al. 1992) are as follows: forward: AAACTAGGATTAGATACCCTATTAT, and reverse: AAGAGCGACGGGCGATGTGT. *Wolbachia wsp* gene primers (Braig et al. 1998, Jeyaprakash and Hoy 2000) are as follows: forward: TGGTCCAATAAGTGATGAAGAAACTAGCTA, and reverse: AAAAATTAAACGCTACTCCAGCTTCTGCAC.
